# 101. Impact of an Integrated Tele-Antimicrobial Stewardship Program at a Rural Community Hospital

**DOI:** 10.1093/ofid/ofab466.303

**Published:** 2021-12-04

**Authors:** Sui Kwong Li, Erin K McCreary, Erin K McCreary, Tina Khadem, Nancy Zimmerman, Sarah Burgdorf, Nupur Gupta, Kate Gass, Gary S Fisher, James W Backstrom, Robin L Portman, Sara Schwarz, Kimberly Schultz, Jenessa Heller, Kris Bearer, Jayne Schreckengost, Jennifer Prazenica, John Mellors, Rima Abdel-Massih, Rima Abdel-Massih, J Ryan Bariola

**Affiliations:** 1 UPMC, Pittsburgh, Pennsylvania; 2 University of Pittsburgh, UPMC, Pittsburgh, PA; 4 Armstrong Center for Medicine & Health, Kittanning, Pennsylvania; 5 University of Pittsburgh, Pittsburgh, Pennsylvania

## Abstract

**Background:**

Small hospitals in the US may lack access to infectious diseases (ID) expertise despite similar rates of antimicrobial use and drug-resistant bacteria as larger hospitals. A tele-antimicrobial stewardship program (TASP) is a force multiplier, expanding access to specialty care, training, and guidance on appropriate resource utilization. Data on the impact of TASPs in community or rural inpatient settings is limited.

**Methods:**

We established a TASP at a 160-bed hospital in Armstrong County, PA (population < 5000) in September 2020. Tele-ID consult services were already being used (Figure 1). A non-local ID pharmacist or ID physician performed prospective audits and provided feedback with 1 local pharmacist on a 30-minute video conference call daily. At TASP implementation, all patients receiving intravenous (IV) fluoroquinolones, metronidazole, and azithromycin were reviewed. Figure 1 shows the additional support following TASP implementation, including addition of ceftriaxone, carbapenems, IV vancomycin, and tocilizumab to daily reviews. A patient monitoring form was developed to track interventions and the local pharmacists were trained in documentation. Table 1 lists other TASP features implemented.

Figure 1. TASP Timeline

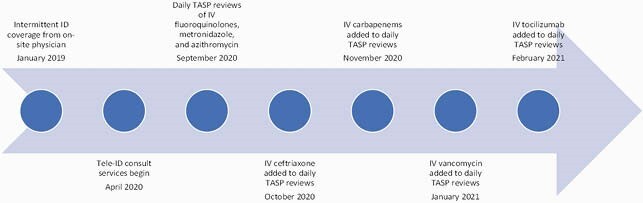

Table 1. TASP Accomplishments

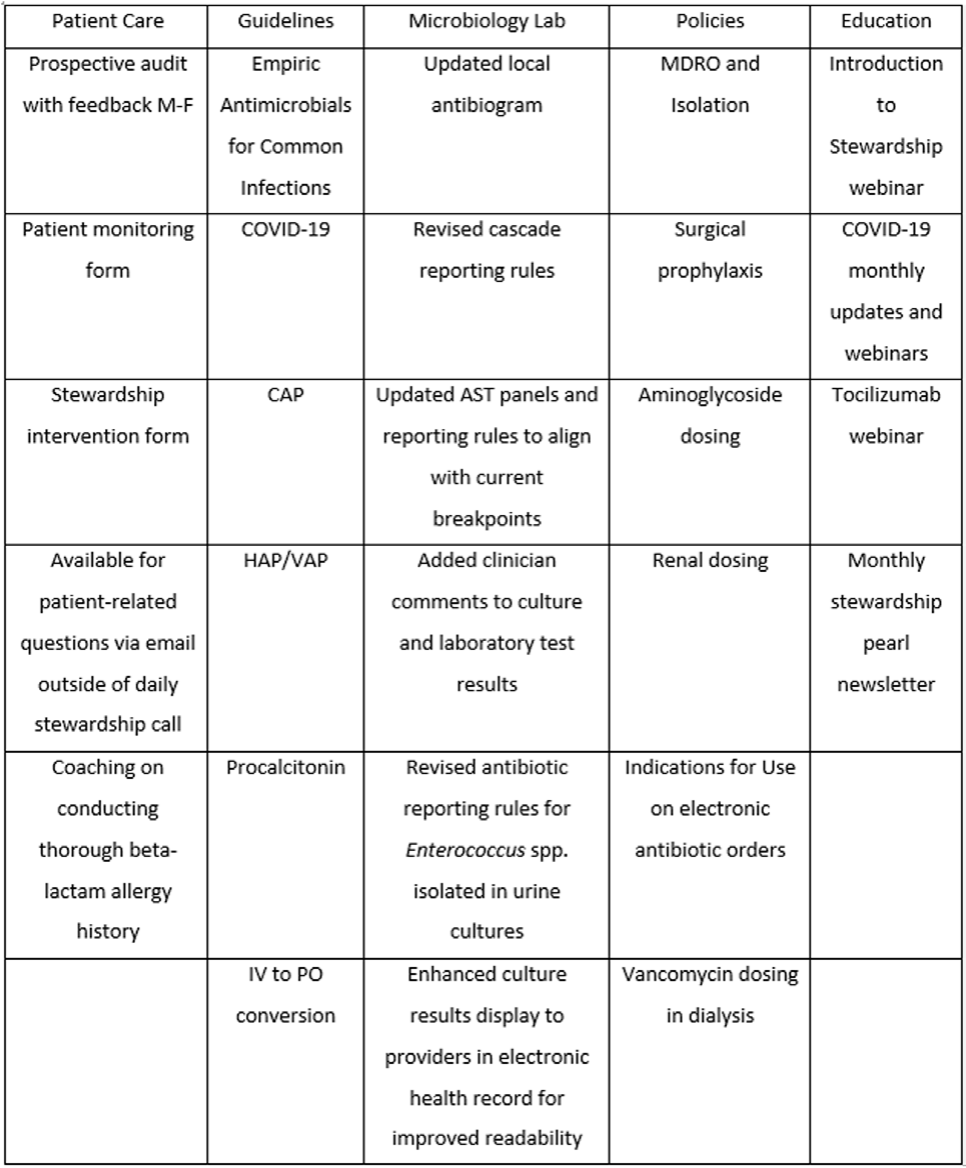

**Results:**

From 09/01/2020 to 04/30/2021, 304 stewardship opportunities were identified and 77% of interventions were accepted. Recommending a duration of therapy was accepted most frequently (93.5%) and de-escalation of therapy least frequently (69.6%) (Table 2). Recommending an ID consultation or diagnostic testing was always accepted but only comprised 6.2% of all interventions. Daily calls involved an average of 5 patient reviews. Monthly antimicrobial use declined on average from 673 DOT (days of therapy)/1000 PD (patient days) to 638 DOT/1000 PD (Figure 2). Daily calls were cancelled on 31/166 weekdays (18.7%) due to staffing shortages.

Table 2. TASP Interventions (9/2020 - 4/2021)

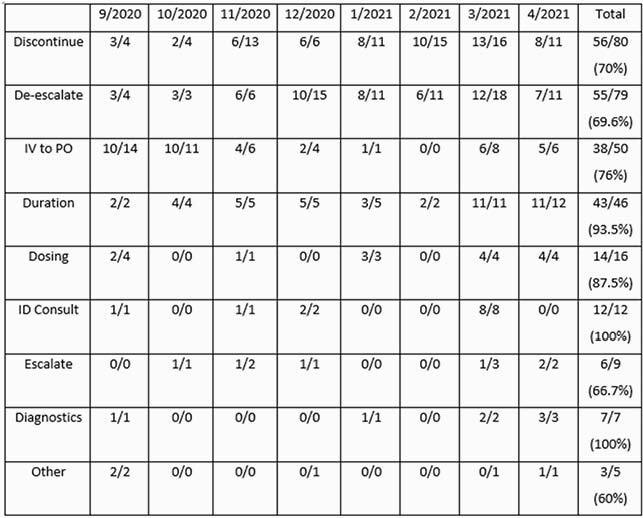

Figure 2. Monthly Antimicrobial Use in Days of Therapy (DOT) per 1000 Patient Days (4/2019 - 5/2021)

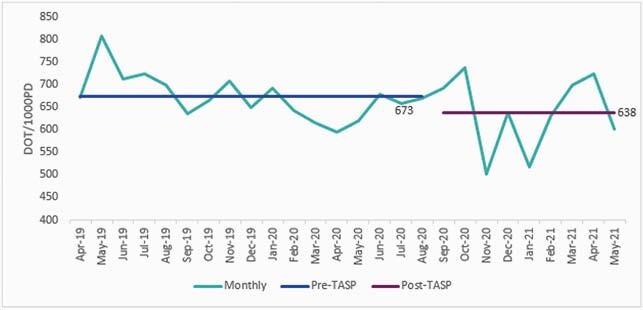

**Conclusion:**

Implementation of TASP in a community hospital resulted in a high percentage of accepted stewardship interventions and lower antimicrobial usage. Success is dependent on robust educational efforts, establishing strong relationships with local providers, and involvement of key stakeholders. Lack of dedicated stewardship time for local pharmacists is a very significant barrier.

**Disclosures:**

**Erin K. McCreary, PharmD, BCPS, BCIDP**, **AbbVie** (Consultant)**Cidara** (Consultant)**Entasis** (Consultant)**Ferring** (Consultant)**Infectious Disease Connect, Inc** (Other Financial or Material Support, Director of Stewardship Innovation)**Merck** (Consultant)**Shionogi** (Consultant)**Summit** (Consultant) **Erin K. McCreary, PharmD, BCPS, BCIDP**, AbbVie (Individual(s) Involved: Self): Consultant; Cidara (Individual(s) Involved: Self): Consultant; Entasis (Individual(s) Involved: Self): Consultant; Ferring (Individual(s) Involved: Self): Consultant; Infectious Disease Connect, Inc (Individual(s) Involved: Self): Director of Stewardship Innovation, Other Financial or Material Support; Merck (Individual(s) Involved: Self): Consultant; Shionogi (Individual(s) Involved: Self): Consultant; Summit (Individual(s) Involved: Self): Consultant **Tina Khadem, PharmD**, **Infectious Disease Connect, Inc.** (Employee) **Nancy Zimmerman, RN, BSN**, **I’d connect** (Employee) **John Mellors, MD**, **Abound Bio, Inc.** (Shareholder)**Accelevir** (Consultant)**Co-Crystal Pharma, Inc.** (Other Financial or Material Support, Share Options)**Gilead Sciences, Inc.** (Advisor or Review Panel member, Research Grant or Support)**Infectious DIseases Connect** (Other Financial or Material Support, Share Options)**Janssen** (Consultant)**Merck** (Consultant) **Rima Abdel-Massih, MD**, **Infectious Disease Connect** (Employee, Director of Clinical Operations) **Rima Abdel-Massih, MD**, Infectious Disease Connect (Individual(s) Involved: Self): Chief Medical Officer, Other Financial or Material Support, Other Financial or Material Support, Shareholder **J Ryan. Bariola, MD**, **Infectious Disease Connect** (Other Financial or Material Support, salary support)

